# Expression profile of intestinal stem cell markers in colitis-associated carcinogenesis

**DOI:** 10.1038/s41598-017-06900-x

**Published:** 2017-07-26

**Authors:** Hye Sung Kim, Cheol Lee, Woo Ho Kim, Young Hee Maeng, Bo Gun Jang

**Affiliations:** 1 Department of Pathology, Jeju National University School of Medicine, Jeju, 690-767 Korea; 2 Department of Pathology, Seoul National University College of Medicine, Seoul, 110-799 Korea

## Abstract

The intestinal epithelium has two distinct two stem cell populations, namely, crypt base columnar (CBC) cells and +4 cells. Several specific markers have been identified for each stem cell population. In this study, we examined the expression profiles of these markers in colitis-associated carcinogenesis (CAC) to investigate whether they can be used as biomarkers for the early detection of dysplasia. The expression of intestinal stem cell (ISC) markers was measured by real-time polymerase chain reaction during CAC that was induced by azoxymethane and dextran sodium sulfate treatment. CBC stem cell markers increased continuously with tumor development, whereas a +4 cell expression profile was not present. CBC stem cell population was suppressed in the acute colitis and then expanded to repopulate the crypts during the regeneration period. Notably, RNA *in situ* hybridization revealed that all dysplasia and cancer samples showed increased expression of CBC stem cell markers in more than one-third of the tumor height, whereas regenerative glands had CBC stem cell markers confined to the lower one-third of the crypt. These results suggest that CBC stem cell markers could be a useful tool for the early detection of colitis-induced tumors.

## Introduction

Inflammatory bowel diseases (IBD) are chronic inflammatory disorders of the intestinal tract that affect millions of people around the world^1, 2^. The two main types of IBD are ulcerative colitis and Crohn’s disease. Management of IBD has so far relied on immunosuppressive therapies, antibiotics, and biological agents targeting mainly the pro-inflammatory tumor necrosis factor (TNF) pathway^3, 4^. One of the consequences of chronic inflammation is the promotion of tumorigenesis; as such, IBD patients have a higher risk of developing colitis-associated colorectal cancer with an odds ratio of approximately 3^5–8^. In colitis-associated carcinogenesis (CAC), a dysplastic precursor lesion arises in colitic mucosa and progresses through various grades of dysplasia^9^. Although the molecular pathogenesis of CAC shares many features with sporadic colorectal cancer, there are differences with respect to the timing and frequency of some alterations that occur during the dysplasia-carcinoma sequence^10^. For example, in CAC, mutations in *TP53* or a loss of p53 function are observed early in disease progression, whereas a functional loss of APC is a late event^9^.

Two models of intestinal stem cell (ISC) identity have historically competed over the past four decades^11^. The ‘stem cell zone model’ suggests that the crypt base columnar (CBC) cells are the resident stem cells^12^, whereas the ‘+4 model’ proposes that stem cells reside immediately above the Paneth cells^13^. Over the last decade, remarkable advances have been made in the ISC research, and specific markers for these two candidate stem cell populations have been proposed through *in vivo* lineage tracing. For instance, Lgr5 (Leu-rich repeat containing G protein-coupled receptor 5) was identified as a selective marker of CBC cells, and these Lgr5^+^ CBC cells were demonstrated to be self-renewing, multipotent adult intestinal stem cells^14^. Additional markers for CBC stem cells include Achaete-Scute homologue 2 (Ascl2)^15^, SPARC-related modular calcium-binding 2 (Smoc2)^16^, Prominin 1(Prom1)^17^, Musashi homologure 1 (Msi1)^18^, Olfactomedin 4 (Olfm4)^19^, and EphB2^20^. Of these, subsequent studies found that the expression of Prom1^21^, Msi1^22^, Olfm4^23^, and Ephb2^23^ extends to the transit amplifying cells. Markers of +4 stem cell population are Bmi1, Homeodomain-only (Hopx), Leu-rich repeats and immunoglobulin-like domains 1 (Lrig1), and telomerase reverse transcriptase (Tert)^11^. Doublecortin-like and CAM kinase-like 1(Dclk1) was originally reported to be a potential +4 stem cell marker, however more recent studies revealed that Dclk1 does not mark normal stem cells, but instead marks differentiated cells such as tuft cells. It should be noted that not all ISC markers have been confirmed by lineage tracing both in small intestine and colon, and some ISC markers such as Smoc2 and Hopx even remain unclear whether they are expressed in colon. The previous results on the lineage tracing and expression patterns of mouse ISC markers in small intestine and colon are summarized in Supplementary Table S1.

Adenomas have been shown to originate from Lgr5^+^ and Bmi1^+^ stem cells in mouse models of tumorigenesis mediated by the Wnt/β-catenin pathway^24, 25^. Most sporadic colorectal cancers in humans also arise from the mutations in the adenomatous polyposis coli (*APC*) gene, and overexpression of stem cell markers has been demonstrated in the sporadic colorectal tumors^23, 26, 27^. However, there is no direct evidence that these stem cells give rise to colitis-associated colorectal cancers, and the expression of ISC markers in CAC has not been thoroughly studied. Therefore, in this study, we aimed to reveal the expression patterns of the ISC markers during CAC using an AOM/DSS colitis-induced colon cancer model to identify ISC markers that could be used for the early detection of dysplasia.

## Results

### Expression of CBC stem cell and +4 stem cell markers in CAC

**Figure 1:**
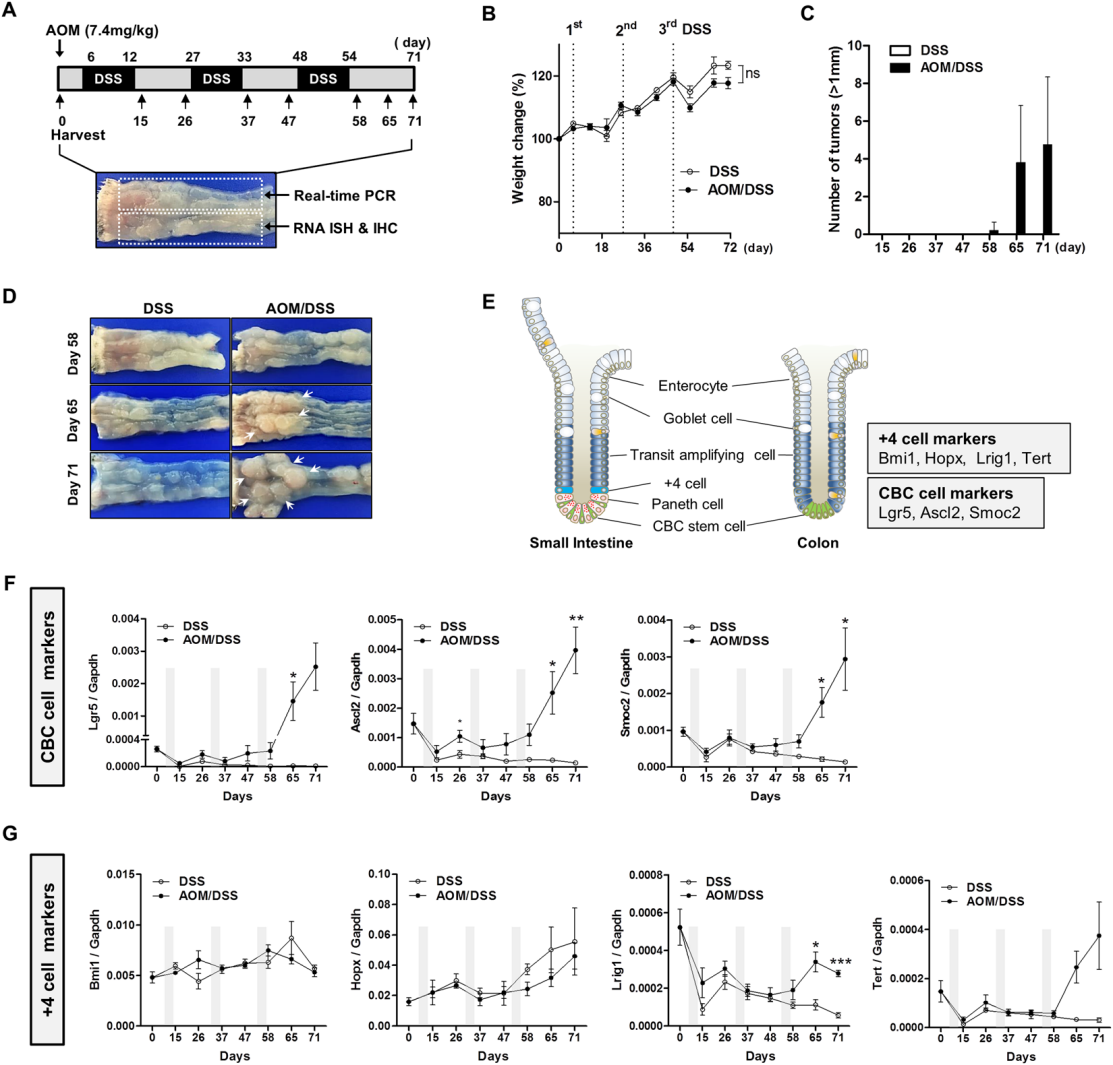
Expression of crypt base columnar (CBC) stem cell and +4 cell markers in colitis-associated carcinogenesis (CAC). (**A**) Schematic diagram of azoxymethane and dextran sodium sulfate (AOM/DSS)-induced colitis-associated colon cancer model. At the days indicated by arrows, mice (DSS: n = 4, AOM/DSS: n = 5) were sacrificed for analysis. (**B**) Body weight changes. Body weight at day 0: 100%. Data represent the mean ± SEM (DSS: n = 28, AOM/DSS: n = 35 at day 0). (**C**) Number of tumors (>1 mm in size) grossly detected in the colon. (**D**) Representative photos of colons harvested at day 58, 65, and 71. Arrows indicate visible tumors. (**E**) Diagram of CBC stem cells and +4 stem cells in the crypt, and their molecular markers. (**F** and **G**) Real-time PCR analysis for CBC stem cell markers; Lgr5, Ascl2, and Smoc2 and +4 stem cell markers; Bmi1, Hopx, Lrig1, and Tert in CAC. Data represent the means ± SEM. (DSS: n = 4, AOM/DSS: n = 5). SC, stem cell; ns, not significant, SEM: standard error of the mean. *P < 0.05; **P < 0.01; ***P < 0.001.

To investigate the expression profiles of ISC markers over CAC, mice were treated with AOM and three cycles of DSS (AOM/DSS group). For the control group (DSS-only group), mice were treated with PBS without AOM followed by the same DSS treatment schedule (Fig. 1A). Before and after each DSS treatment, the distal colon was harvested and subjected to real-time polymerase chain reaction (PCR) analysis and histologic examination. At the last harvest (day 71), body weight loss was slightly higher in the AOM/DSS group (117.7 ± 1.8, n = 7) compared to that in the control group (123.3 ± 1.3, n = 3), but this was not statistically significant (Fig. 1B, *P* = 0.10). As expected, only AOM/DSS group mice started to develop grossly visible colon tumors after the third cycle of DSS treatment, the majority of which were found in the distal colon (Fig. 1C). Representative photographs of colons harvested at day 58, 65, and 71 in control and AOM/DSS group mice are shown in Fig. 1D.

It is believed that two types of intestinal stem cell population exist; CBC cells at the bases of crypts and +4 cells at the +4 position. Several specific molecular markers have been identified for each population, specifically, Lgr5, Ascl2, and Smoc2 for CBC stem cells and Bmi1, Hopx, Lrig1, and Tert for +4 stem cells (Fig. 1E). We measured the mRNA expression of these markers over the course of carcinogenesis. Remarkably, all CBC stem cell markers, Lgr5, Ascl2, and Smoc2, showed the same expression patterns (Fig. 1F). After the first DSS treatment, CBC stem cell markers substantially decreased and then increased during the recovery period although not to the level of that of normal mucosa; this was similarly observed in both the control and AOM/DSS groups. However, after the second DSS treatment, the expression of CBC stem cell markers in the AOM/DSS group continued to dramatically increase, whereas that in the control group continued to decline. In contrast, of the +4 stem cell markers, Bmi1 and Hopx expression showed no significant difference between the AOM/DSS and control groups (Fig. 1G). Although, after the third cycle of DSS treatment, Lrig1 and Tert expression was higher in the AOM/DSS group than in control, expression levels were extremely low or even lower than that in the normal colonic mucosa.

### Expression of tuft cell and progenitor cell markers in a colitis associated colon cancer

**Figure 2:**
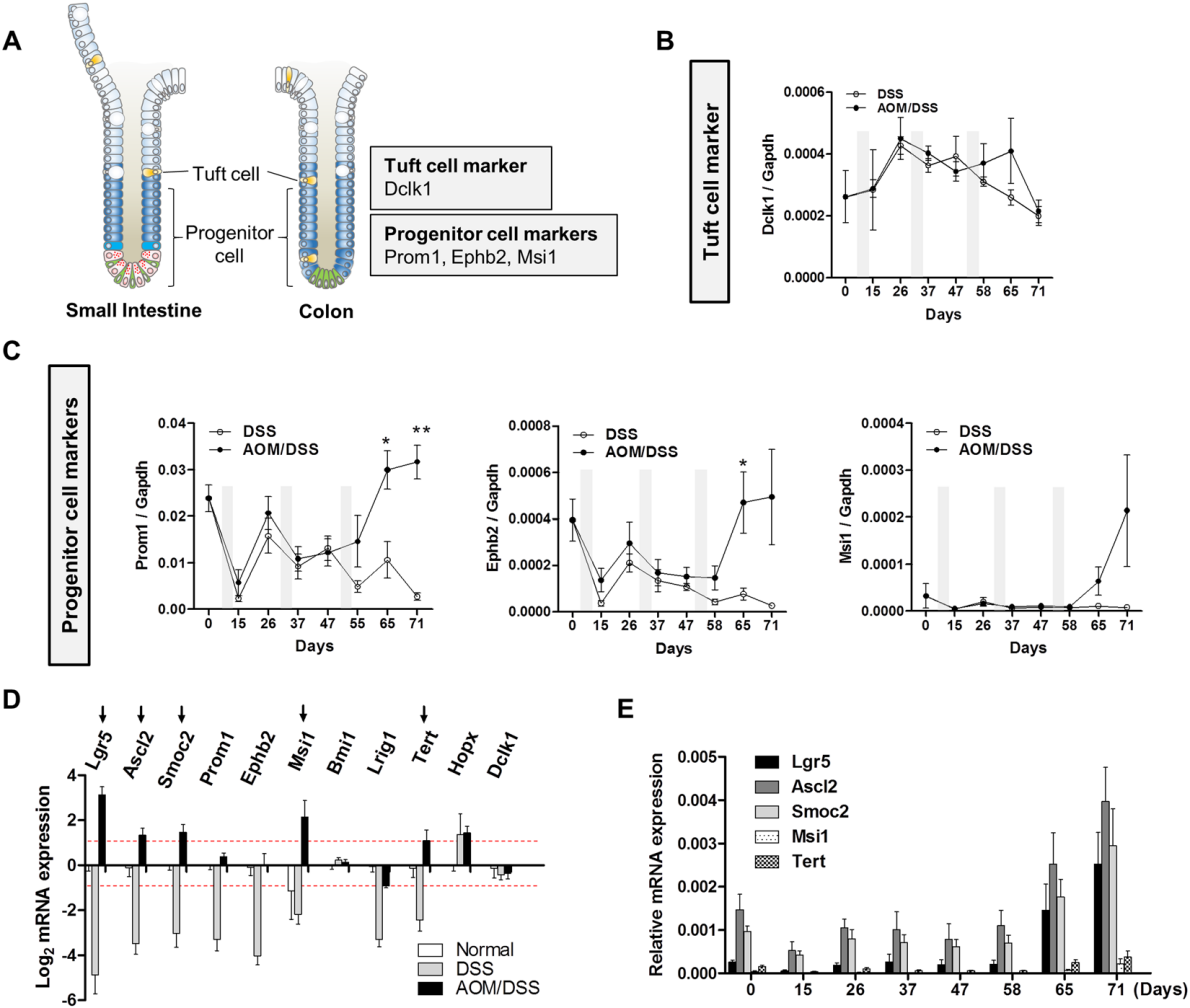
Expression of tuft cell and progenitor cell markers in colitis-associated carcinogenesis (CAC). (**A**) Diagram of tuft cell and progenitor cell markers in the crypt. (**B** and **C**) Real-time PCR analysis of tuft cell marker Dclk1 and progenitor cell markers Prom1, Ephb2, and Msi1. Data represent the means ± SEM. (DSS: n = 4, AOM/DSS: n = 5). (**D**) Relative mRNA expression of ISC markers in control and azoxymethane and dextran sodium sulfate (AOM/DSS) groups at day 71 compared to that in the normal colonic mucosa. Arrows indicate the markers that are expressed at two-fold higher levels in the AOM/DSS group and two-fold lower levels in the DSS group compared to expression in the normal colonic mucosa. Red dotted lines indicate the threshold. (**E**) Comparison of relative mRNA expression of five markers in CAC. SEM, standard error of the mean. *P < 0.05; **P < 0.01.

Next, we assessed the expression of the so-called tuft cell marker, Dclk1 and progenitor cell markers, Prom1, Ephb2, and Msi1 (Fig. 2A). As mentioned earlier, because Prom1, Msi1, and Ephb2 have broader expression in transit amplifying cells, they were classified as progenitor cell markers in this study even though they were originally proposed to be CBC stem cell markers. Olfm4 also was suggested to be a surrogate marker for Lgr5^19^, and we previously showed that OLFM4 is a progenitor marker in the human colon^23^. However, as Olfm4 is not expressed in the mouse colon^19^, it was not included in this study. Dclk1 showed no significant expression pattern and no difference in the expression levels between the AOM/DSS and control groups (Fig. 2B). Prom1 and Ephb2 showed a similar expression pattern as CBC stem cell markers, but its expression level in the late phase (after 58 days) was not substantially increased compared to that in the normal colon (Fig. 2C). Msi1 expression was higher in the tumor phase, but its expression level was very low (Fig. 2C).

To select markers associated with tumor development, we compared the expression level of each stem cell marker at the last time point of colon harvesting (day 71) to that in the normal colonic mucosa (day 0). Five markers including Lgr5, Ascl2, Smoc2, Msi1, and Tert were found to increase more than two-fold in the AOM/DSS group and decrease more than two-fold in the control group (Fig. 2D). By comparing the expression of these five markers, we chose three CBC stem cell markers, Lgr5, Ascl2, and Smoc2, for RNA *in situ* hybridization (ISH) analysis to confirm their localization and expression histologically because Msi1 and Tert expression levels were too low for use diagnostically (Fig. 2E).

### Expansion of CBC stem cell population at the regenerating glands during the recovery period

**Figure 3:**
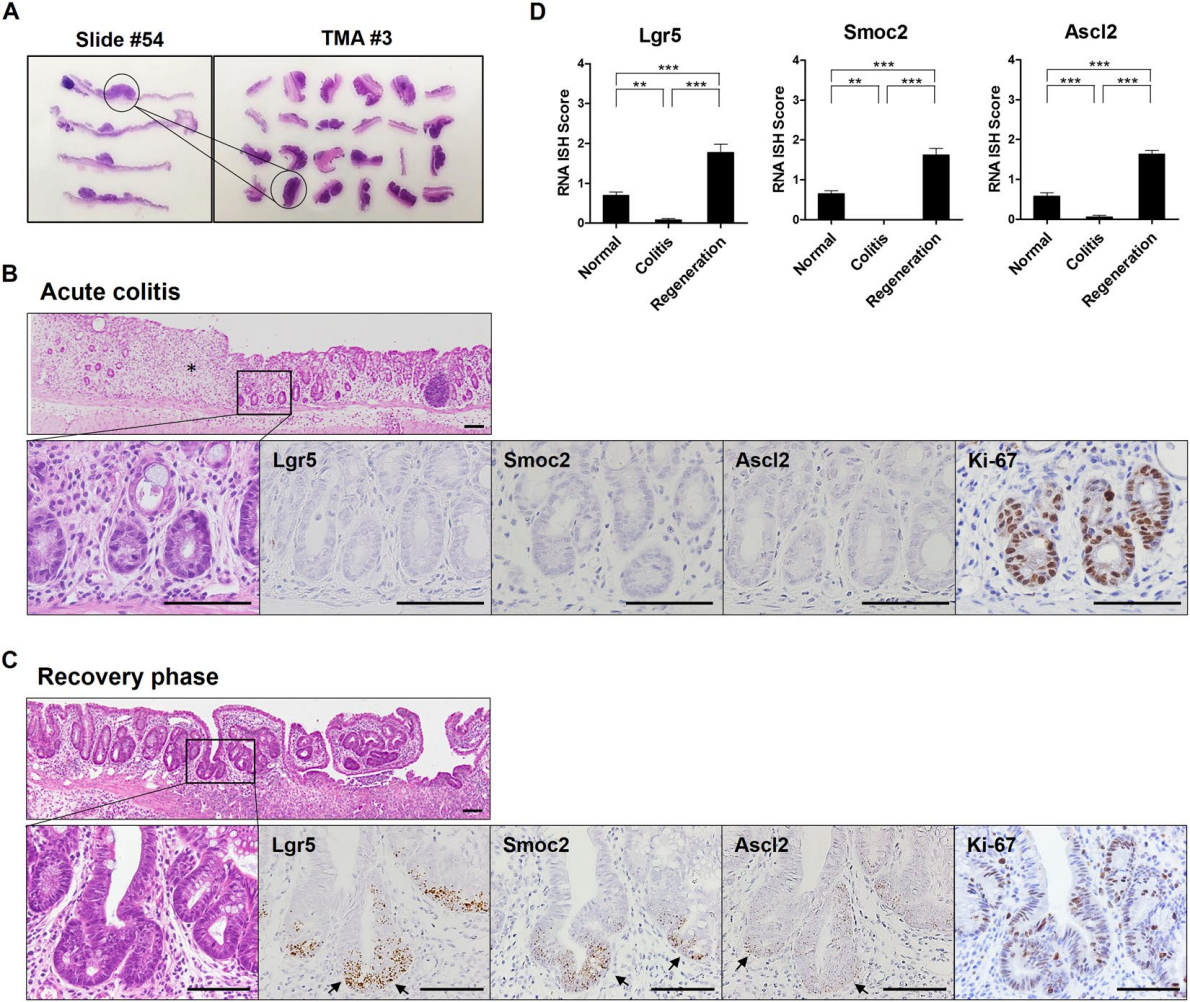
Crypt base columnar (CBC) stem cell marker expression during regeneration after acute colitis. (**A**) Construction of three tissue microarrays from 56 hematoxylin and eosin-stained slides, containing 72 representative cores. (**B**) Representative images of acute colitis after the first DSS treatment (day 15) with reduced expression of CBC stem cell markers. RNA *in situ* hybridization (ISH) for Lgr5, Smoc2, and Ascl2 and immunohistochemistry for Ki-67. Asterisk indicates crypt loss in the lamina propria. Scale bar: 50 μm. (**C**) Representative images of regenerative glands (day 26) with increased expression of CBC stem cell markers at the base of the crypts. Arrows indicate stem cell marker-expressing cells. (**D**) Expression of CBC stem cell markers in normal mucosa, acute colitis, and during regeneration. RNA ISH score was assessed in 38 to 67 colon crypts. Data represent the means ± SEM. (N: normal, n = 2; C: colitis, n = 3; R: regeneration, n = 4). SEM, standard error of the mean. **P < 0.01; ***P < 0.001.

Half of the distal colon tissue was obtained from each sacrificed mouse, from which 56 FFPE blocks and H&E-stained slides were generated. Through histologic examination, representative lesions were selected and three tissue microarrays (TMAs) with 72 cores were constructed containing 29 foci of regenerative glands (RG), and 47 tumors including 10 low grade dysplasia (LGD), 20 (high grade dysplasia) HGD, and 17 (adenocarcinoma) ADC samples (Fig. 3A).

Before we focused on the expression of CBC stem cell markers in RG and tumors, we investigated alterations in the CBC stem cell population during regeneration after colitis-induced mucosal damage. After the first DSS treatment (day 15), the colon mucosa showed the acute colitis accompanied by marked crypt destruction and loss, consistent with real-time PCR results showing a decline in the expression of most stem cell markers. The small number of residual glands rarely expressed the CBC stem cell markers at the crypt bases (Fig. 3B). After 10 days of recovery (day 26), regeneration of glands with irregular branching was observed with enhanced expression of CBC stem cell markers at the crypt bases (Fig. 3C and Supplementary Fig. S1). When comparing the RNA ISH scores for crypt bases between normal mucosa, acute colitis, and regenerating mucosa, all CBC stem cell markers were lower in the inflamed mucosa and were higher in the regenerating glands than in the normal colonic mucosa (Fig. 3D). These findings suggest that the CBC stem cell population is suppressed in the inflammatory condition and then expands to repopulate the crypts during the recovery period.

### Localization of CBC stem cell markers in colitis-induced dysplasia and cancers

**Figure 4:**
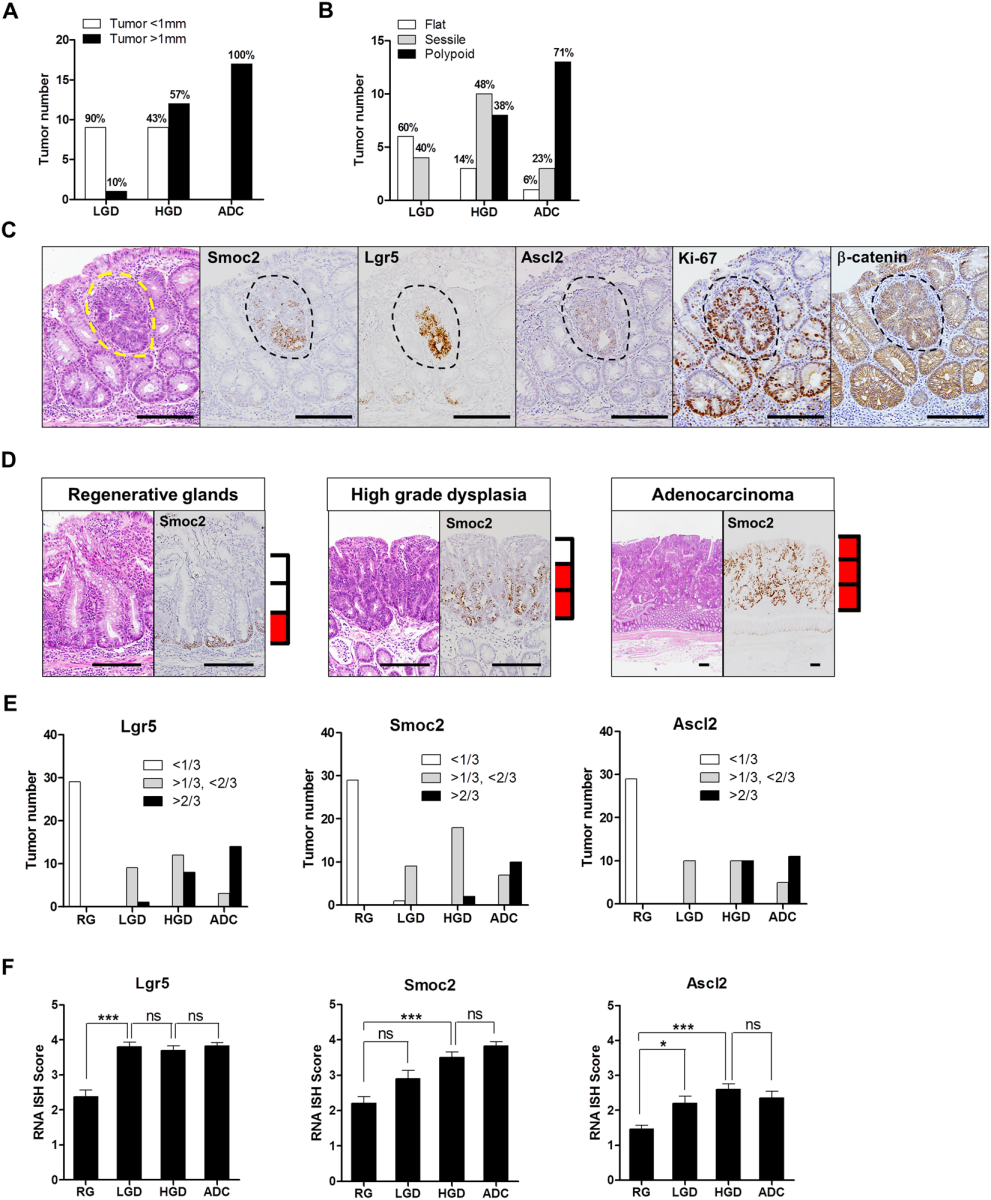
Distribution and intensity of crypt base columnar (CBC) stem cell markers in colitis-associated dysplastic lesions and cancers. (**A**) The number of tumors according to size and histologic grades. (**B**) The number of tumors according to gross appearance and grades. (**C**) RNA *in situ* hybridization (ISH) for Smoc2, Lgr5, and Ascl2 in an aberrant crypt focus indicated by a yellow or black dotted lines. Scale bar: 100 μm. (**D**) Representative images of Smoc2 expression with three distribution patterns in RG, HGD, and ADC cases. Red box indicates the area of Smoc2-expressing cells in the crypt or tumor height. (**E**) Distribution of CBC stem cell markers in all RG (n = 29), LGD (n = 10), HGD (n = 20), and ADC (n = 17) cases. <1/3: lower than one-third of the crypt or tumor height; >1/3, <2/3: higher than one-third, but lower than two-thirds of the crypt or tumor height; >2/3: higher than two-thirds of the crypt or tumor height. (**F**) RNA ISH intensity of CBC stem cell marker expression. Data represent the means ± SEM. (RG: regenerative glands, LGD: low grade dysplasia, n = 10; HGD: high grade dysplasia; ADC, adenocarcinoma; ns, not significant; SEM, standard error of the mean. *P < 0.05; ***P < 0.001.

Forty-seven tumors including 10 LGD, 20 HGD, and 17 ADC samples were evaluated according to size and gross appearance. Most (90%, 1 of 10cases) LGD samples were small (less than 1 mm) and all ADCs were over 1 mm (Fig. 4A). Grossly, LGD samples tended to be flat (60%) or sessile (40%), whereas ADCs were mostly polypoid (71%) (Fig. 4B). Aberrant crypt foci (ACF) are thought to be the earliest detectable neoplastic lesions in the colon carcinogenesis model^28^. We also identified a few ACF that expressed increased levels of Smoc2, Lgr5, and Ascl2 with a disorganized distribution pattern (Fig. 4C and Supplementary Fig. S2A).

**Figure 5:**
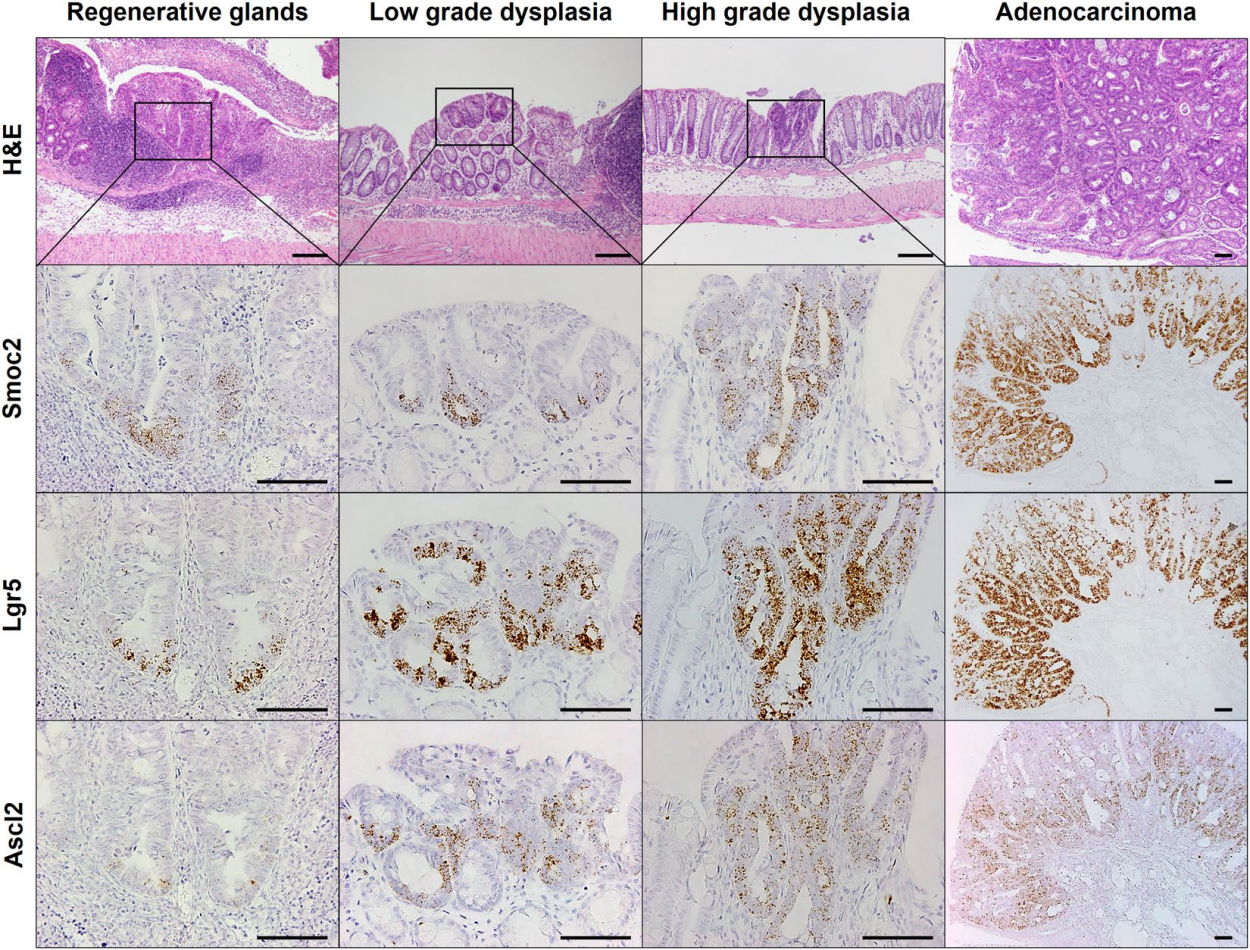
RNA *in situ* hybridization for crypt base columnar (CBC) stem cell markers in colitis-associated dysplasia and cancer. Representative hematoxylin and eosin-stained images of regenerative glands, low grade dysplasia, high grade dysplasia, and adenocarcinoma. RNA *in situ* hybridization (ISH) demonstrates the expression of Smoc2, Lgr5, and Ascl2 in each lesion. Scale bars: 50 μm.

For all RG and tumors in the TMAs, we assessed the expression of CBC stem cell markers with regard to distribution pattern and intensity using RNA ISH. An example of different spatial distribution patterns is shown in Fig. 4D. We found a striking difference in the distribution of CBC stem cell markers between RG and tumors. All RG had CBC stem cell markers restricted to the lower one-third of glands, as normal crypts, whereas all dysplasia samples and ADCs, except one case of LGD, exhibited stem cell marker expression in more than one-third of the tumor height. (Fig. 4E). In most polypoid tumors, the expression of CBC stem cell markers extended to more than two-thirds of the tumor height (Supplementary Fig. S2B). The intensity of RNA ISH was also significantly higher in cases of dysplasia and ADCs than in RG (Fig. 4F). Representative images of CBC stem cell markers in each lesion are shown in Fig. 5. RG often exhibit atypical cytologic and structural features, however, the expression of CBC stem cell markers was confined to the bottom of the glands. In one case of LGD, Smoc2 appeared to be limited to tumor bases, but Lgr5 and Ascl2 showed diffuse and patchy distribution. HGD cases and ADCs expressed CBC stem cell markers in a diffuse manner although the expression of stem cell markers tended to be intense at the tumor bases, indicating a disorganized or loss of stem cell hierarchy with tumor progression. Furthermore, in 5 ulcerative colitis patients, we also clearly detected the same expression patterns of CBC stem cell markers in colitis-associated lesions as those in the mouse model (Supplementary Fig. S3).

### Activation of the Wnt pathway in CAC

**Figure 6:**
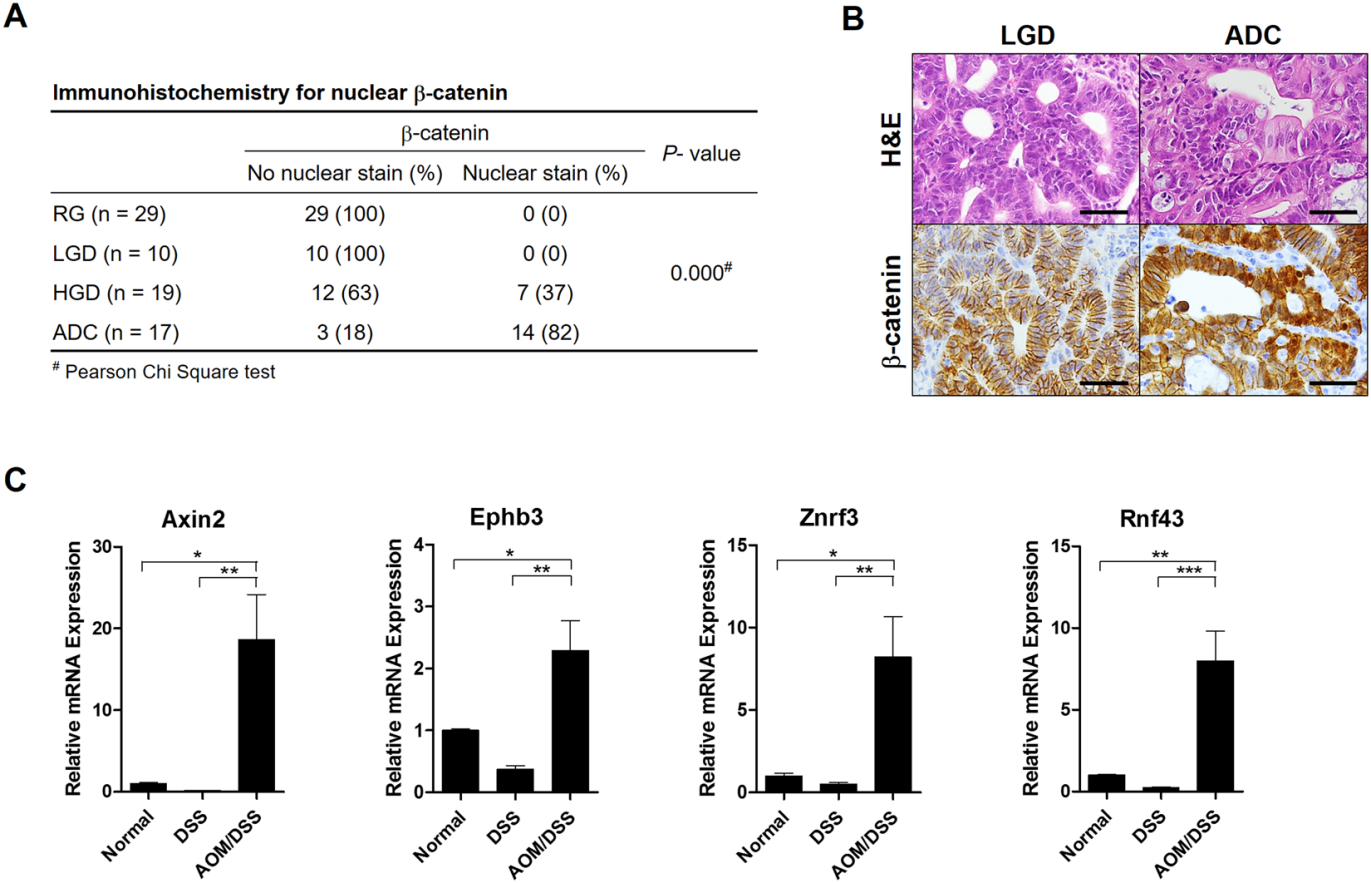
Enhanced Wnt signaling pathway in colitis-associated carcinogenesis. (**A**) Immunohistochemical analysis revealed that nuclear β-catenin staining was negative in all regenerative glands and low grade dysplasia (LGD) samples, but was positive in 37% of high grade dysplasia cases and 82% of adenocarcinomas (ADC). (**B**) Representative pictures of a LGD samples with membranous β-catenin and an ADC with strong cytoplasmic and nuclear β-catenin staining. Scale bar: 25 μm (**C**) Other Wnt-target genes including Axin2, Ephb3, Znrf3, and Rnf43 were upregulated in the azoxymethane and dextran sodium sulfate (AOM/DSS) group compared to expression in the normal and control groups. (Normal: n = 3, DSS: n = 7, AOM/DSS: n = 5). *P < 0.05; **P < 0.01; ***P < 0.001.

Colitis-associated cancers have distinct molecular characteristics different from sporadic colon cancers, one of which is that Wnt pathway activation caused by *APC* mutation, which is an early event in sporadic colon cancer, but occurs at later stages in CAC. Thus, we performed immunohistochemical analysis of β-catenin, as nuclear β-catenin expression represents abnormal activation of the Wnt signaling pathway. Indeed, no RG or LGD samples showed nuclear β-catenin staining, whereas 37% (7 of 19 cases) of HGD cases and the majority of (82%, 14 of 17cases) ADC were positive for nuclear β-catenin (Fig. 6A). Representative images are shown in Fig. 6B, wherein one example of LGD displayed clear membranous staining, whereas one ADC sample showed cytoplasmic and nuclear staining. To further confirm enhanced Wnt signaling in the late stages of CAC, we examined the expression of other Wnt-target genes including Axin2, Ephb3, Znrf3, and Rnf43. Compared to the normal mucosa and the control group, the AOM/DSS group revealed significantly higher levels of all Wnt-target genes (Fig. 6C).

## Discussion

Over decades of intensive study, researchers have successfully deciphered many key aspects of intestinal biology, including the approximate size and location of the adult stem cell population within the intestinal epithelium^11^. With the discovery of robust intestinal stem cell markers by lineage tracing technologies, impressive breakthroughs have been made in understanding how intestinal stem cells interact with their local niche to maintain homeostasis and how they contribute to intestinal cancer initiation and progression. In particular, in the *Apc*-induced tumorigenesis, tumors have been shown to originate from Lgr5^+^ CBC stem cells^24^. However, little is known about the involvement of the stem cell compartment in CAC. In this study, we demonstrated the expression profiles of various ISC markers over the course of CAC using the AOM/DSS model. In addition, by constructing tissue microarrays, we directly visualized the expression of ISC markers in many colitis-associated lesions; this made it possible to analyze the spatial differences of the expression patterns of markers between RG and dysplastic lesions.

To our knowledge, this is the first study to continuously measure the mRNA expression of ISC markers during the entire course of inflammation-induced colon carcinogenesis. Among many candidate ISC markers reported to date, we selected 11 well-established markers, seven of which have been directly proven to be ISC markers by *in vivo* lineage tracing; these included Lgr5, Lrig1, and Tert in both the small intestine and colon: Smoc2, Bmi1, Hopx, and Prom1 only in the small intestine (Supplementary Table S1). Remarkably, CBC stem cell otmarkers showed a distinct expression pattern, specifically, gradual increase with tumor development. We confirmed these results by RNA *in situ* hybridization, showing that both the regenerating mucosa and dysplastic lesions strongly expressed CBC stem cell markers. In contrast, markers for +4 stem cells, tuft cells, and progenitor cells did not show significant expression patterns. Even though the expression of Tert and Msi1 in the AOM/DSS group was higher than that in the control group in the late phase, their expression levels were much lower than CBC stem cell markers, which was consistent with the results from RNA ISH analysis (Supplementary Fig. S4).

Although we divided 11 ISC markers into four categories, namely, CBC stem cell, +4 stem cell, tuft cell, and progenitor cell markers, based on the published reports, many recent studies have casted doubt on the validity of these markers for specific intestinal stem cell population. For example, Munoz *et al*. showed that four +4 stem cell markers, Bmi1, Hopx, Lrig1, and Tert are also expressed in Lgr5^+^ CBC cells and exhibit a broad expression pattern throughout the crypt axis in the small intestine with no evidence for specific enrichment of these markers outside the Paneth cell/ Lgr5 stem cell zone.^16^ Dclk1, Prom1, Ephb2, and Msi1 were shown to have a broad expression gradient along the crypt^29^. These findings imply that many stem cell markers cannot be used as a specific marker for intestinal stem cell population^11, 16, 29^. This might in part explain why +4 stem cell markers did not show significant expression patterns in CAC. In contrast, Lgr5, Ascl2, and Smoc2 are known to be exclusively expressed in CBC stem cells^11^. Our results support the notion that CBC stem cell markers represent a very specific stem cell population involved in the CAC.

DSS treatment in the drinking water induced severe colitis, leading to significant crypt loss (Fig. 3B). As a result, most ISC markers decreased substantially after the first DSS treatment. In this situation, intestinal stem cells are expected to proliferate rapidly and rebuild crypt architecture during the recovery period. Indeed, real-time PCR results showed that the expression of 8 of 11 ISC markers increased at 11 days of DSS recovery. In particular, RNA ISH demonstrated a dramatically increased CBC stem cell population expressing Lgr5, Smoc2, and Ascl2 at the base of regenerating mucosa, consistent with a previous report showing that Lgr5 stem cells reappeared at day 5 of DSS recovery, with normal levels attained by day 6 of recovery^30^. These findings suggest that Lgr5 + CBC stem cells might have a crucial role in regeneration after colitis-induced damage. It is also reasonable to speculate that repeated colitis/regeneration cycles might increase the risk of tumor development by expanding the CBC stem cell population because tumors originate from CBC stem cells that are vulnerable to transformation as we previously suggested in the gastric tumorigenesis^31^.

Surveillance colonoscopy is currently the most widely used method to detect dysplasia and cancer in IBD patients^32^. Multiple biopsies are necessary to adequately screen the entire length of the colon. However, RG and dysplasia can be often difficult to distinguish by histology alone although regenerative changes are identified at the base of the crypts and exhibit surface maturation, whereas dysplasia involves the entire crypt and surface epithelium. Several biomarkers have been suggested for identifying and distinguishing dysplastic lesions in IBD. For instance, the combination of p53 and Ki-67 is a useful to differentiate dysplastic lesions from inflammatory reactive changes in IBD biopsy specimens^33^, and the combination of p53, Ki-67, and β-catenin could be a practical panel to help distinguish dysplasia-associated dysplasia from sporadic adenomas^34^. More recently, the combination of p53 and a-Methylacyl coenzyme A racemase (AMACR) has been suggested to be a tool for confirming dysplasia in IBD^35^. In this study, our results suggest CBC stem cell markers to be additional biomarkers for identifying dysplastic lesions. For diagnostics, the distribution pattern appears to be more promising rather than the expression intensity because no RG expressed CBC stem cell markers in more than one-third of the crypt height, regardless of the degree of cytologic and structural atypia, whereas all dysplasia cases and cancers, except one case, showed CBC stem cell markers extending over one-third of the tumor height. Recently, it has been shown that most (75%) tumors induced by the AOM/DSS protocol in *Lgr5-eGFP* reporter mice express Lgr5, and which can be detected by confocal laser endomicroscopy, suggesting that Lgr5 could be a molecular marker for the early detection of colitis-associated tumors^36^. Based on our results, not only Lgr5, but also Smoc2 and Ascl2, could be used for the same purpose, or these markers could be combined together to increase sensitivity to identify early dysplasia. In particular, for flat lesions that are more commonly found in colitis-associated tumors than in sporadic colon tumors, these markers can be helpful to detect very early IBD-associated dysplasia. Of progenitor markers, we also observed a strong Prom1 expression in ACF and cancers by RNA ISH. However, high Prom1 expression was also detected diffusely in the surface epithelial cells of normal crypts and RG, indicating that Prom1 is less likely to be useful as a diagnostic marker (Supplementary Fig. S4). Notably, we found that the samples of regenerative glands and dysplasia from 5 ulcerative colitis patients showed the same expression patterns of CBC stem cell markers as in the mouse samples (Supplementary Fig. S3). Additional studies with a larger number of human IBD samples are required to validate these results, and the generation of reliable antibodies to CBC stem cell markers for endoscopic surveillance is demanded.

Abnormally enhanced Wnt signaling, via APC mutations, is a key molecular event in the early stages of sporadic colorectal carcinogenesis, whereas, in the CAC, *APC* mutations occur in the late stages. Indeed, we did not find nuclear β-catenin staining in the early lesions such as ACF or LGD samples. In contrast, we observed a high rate of nuclear β-catenin in HGD and ADC cases, with elevated expression of other Wnt-target genes such as Axin2, Ephb3, Rnf43, and Znrf3 in the late stages. Interestingly, however, the early lesions without nuclear β-catenin strongly expressed CBC stem cell markers, Lgr5, Smoc2, and Ascl2, which are also Wnt-target genes. Therefore, it seems that the expression of CBC stem cell markers in ACF and LGD samples does not rely on upregulated Wnt signaling activity via *Apc* mutations. In fact, upregulation of CBC stem cell markers at the base of RG during the recovery does not require the mutations in the Wnt signaling pathway. Thus, it is likely that physiologic Wnt pathway activation is sufficient to induce and maintain the expression of CBC stem cell markers in the RG and early dysplastic lesions.

In summary, the present study revealed the expression profiles of a number of ISC markers during CAC using the AOM/DSS model. CBC stem cell markers, but not +4 stem cell markers, gradually increased as tumors developed with repeated colitis. RNA ISH confirmed that all dysplastic lesions and cancers expressed high levels of CBC stem cell markers. Notably, these tumors can be distinguished from RG based on the fact that CBC stem cell markers are expressed in over one-third of the tumor height, whereas it was confined to the lower one-third of crypts in RG. Collectively, these observations suggest that CBC stem cell markers are closely associated with the development of colitis-associated colon cancer and could be used as additional diagnostic markers for the early detection of colitis-induced tumors.

## Materials and Methods

### Mice and human samples

All experiments used 8- to 10-week-old C57BL/6 male mice that were purchased from OrientBio (Seongnam, Republic of Korea) and maintained at the Animal Research Facility at Jeju National University School of Medicine under specific pathogen-free conditions. Animal experiments were performed based on the Institutional guideline of Jeju National University for animal use and care. Formalin-fixed and paraffin-embedded (FFPE) human tissue samples from five ulcerative colitis (UC) patients were obtained from Jeju National University Hospital. Histopathological classification of each patient sample was independently carried out by two gastrointestinal pathologists (YHM and BGJ). All experimental protocols were approved by the Institutional Review Board of Jeju National University, and all methods were carried out in accordance with approved guidelines.

### Induction of CAC and harvesting of colon tissue

Mice (n = 32) were intraperitoneally injected with 7.4 mg/kg body weight of AOM (Sigma-Aldrich, St Louis, MO, USA) dissolved in phosphate-buffered saline (PBS). Five days after AOM administration, 1% DSS (MP Biomedicals, Santa Ana, CA, USA) was added to the animals’ drinking water for 1 week, and then water without DSS was provided for 2 weeks (first cycle). Each cycle consisted of 1 week of DSS treatment and a 2-week of recovery period. The control group (n = 27) followed the same protocol excluding the AOM injection on day 0. Body weight was measured once every week. Mouse colon tissues were harvested before and after each DSS treatment throughout the study duration (Fig. 1A). Upon opening the colon, the entire luminal surface was observed and tumors (greater than 1 mm in diameter) were enumerated. Approximately 1 cm of distal colon was bisected longitudinally; half was stored in RNA*later*® stabilization solution (Ambion, Austin, TX, USA) for RNA analysis and the other half was fixed in 4% paraformaldehyde in neutral buffer solution for paraffin embedding.

### Real-time PCR analysis

Total RNA (2 μg) extracted using TRIZOL reagent (Invitrogen, Carlsbad, CA, USA) was subject to reverse-transcription with oligo-dT primers and the GoScript reverse transcription system (Promega, Madison, Wisconsin, USA). PCR reactions were performed with Premix EX Taq (Takara bio, Shiga, Japan) according to the manufacturer’s recommendations, and the cycling conditions were followed: initial denaturation for 30 s at 95 °C, followed by 40 cycles of 95 °C for 1 s and 60 °C for 20 s in a StepOne Plus real-time PCR system (Applied Biosystems, Foster City, CA, USA). The TaqMan gene expression assays were used as follows: Mm00438890_m1 (Lgr5), Mm01268891_g1 (Ascl2), Mm00491553_m1 (Smoc2), Mm00477115_m1 (Prom1/CD133), Mm01181021_m1 (Ephb2), Mm01203522_m1 (Msi1), Mm03053308_g1 (Bmi1), Mm00558630_m1 (Hopx), Mm00456116_m1 (Lrig1), Mm00436931_m1 (Tert), Mm00444950_m1 (Dclk1), Mm00443610_m1 (Axin2), Mm00802553_m1 (Ephb3), Mm00552558_m1 (Rnf43), Mm01191453_m1 (Znrf3), and Mm99999915_g1 (Gapdh). Gapdh served as the endogenous control.

### Tissue microarray construction

Three TMAs containing 24-cores from control (DSS only) and 48-cores from AOM/DSS groups were constructed. In brief, 18 and 38 FFPE blocks were generated from control and AOM/DSS groups, respectively. Paraffin-embedded tissues were cut into 4-μm sections and stained with hematoxylin and eosin (H&E). Through histologic examination by two independent gastrointestinal pathologists (YHM and BGJ), from one to three representative areas such as RG, LGD, HGD, and ADCs were marked on each slide. The diameter of each core was 4 mm, which is sufficient size to identify any tumors that developed in the colon of AOM/DSS mice, making it possible to examine the expression of ISC markers in the entire tumor. One TMA was also constructed from 5 human UC patient samples, which includes 7 regenerative glands, 2 inflammatory polyps, 2 flat and 3 polypoid low grade dysplasia samples. Core tissue biopsies containing marked areas were obtained from individual FFPE tissues (donor blocks) and arranged in a new recipient paraffin block (tissue array block) using a trephine apparatus (SuperBioChips Laboratories, Seoul, Korea).

### RNA *in situ* hybridization and interpretation

ISH was performed using RNAscope FFPE assay kit (Advanced Cell Diagnostics, Inc., Hayward, CA, USA) as described previously (Jang *et al*.^31^). Briefly, 4-μm tissue sections of TMA are pretreated with heat and protease digestion followed by hybridization with the probe; Lgr5, Smoc2, Ascl2, Msi1, Prom1 and Tert for mouse samples, and LGR5, SMOC2, and ASCL2 for human samples. Then, an HRP-based signal amplification system is hybridized to the probe before color development with 3,3′-diaminobenzeidine tetrahydrochloride (DAB). Positive staining was indicated by brown punctate dots in the nucleus and/or cytoplasm. Expression of ISC markers was quantified according to the manufacturer’s scoring guideline: score 0, no staining or less than one dot per cell; score 1: 1 to 3 dots per cell (visible at x20–40 magnification); score 2: 4 to 10 dots per cell and no or very few dot clusters (visible at x20–40); score 3: >10 dots per cell and fewer than10% positive cells have dot clusters (visible at x20); score 4: >10 dots per cell and >10% of positive cells have dot clusters (visible at x20).

### Immunohistochemistry

Immunohistochemistry was performed on 4-μm TMA sections using a BOND-MAX automated immunostainer and a Bond Polymer Refine Detection kit (Leica Microsystems, Wetzlar, Germany) according to the manufacturer’s instructions. The primary antibody used was anti-Ki-67 (Dako, Carpinteria, CA, USA; MIB-1; 1:100) and anti-β-catenin (Novocastra Laboratories Ltd., Newcastle, UK; 17C2; 1:800). β-catenin staining was considered positive when >10% of the tumor cell nuclei were strongly stained for β-catenin.

### Statistical analysis

Statistical analyses were performed using the PASW 18.0 statistical software program (IBM SPSS Statistics, Chicago, IL, USA) and Prism version 5.0 (GraphPad Software, Inc., San Diego, CA, USA). Between-group comparisons of the real-time PCR data and RNA ISH scores were performed using Turkey’s multiple comparison test. The correlation between nuclear β-catenin positivity and histology was tested using Pearson’s chi-square test. A *P*-value < 0.05 was considered statistically significant.

## Electronic supplementary material

Supplementary Data
