# Supplementary material for: Expression profile of intestinal stem cell markers in colitis-associated carcinogenesis
Source: Sci Rep. 2017 Jul 26;7:6533. doi: 10.1038/s41598-017-06900-x (PMC5529509; doi:10.1038/s41598-017-06900-x)
Supplement: Supplementary file 1 — Supplementary Data [file 41598_2017_6900_MOESM1_ESM.pdf]

Supplementary Information

Expression profiles of intestinal stem cell markers in colitis-associated carcinogenesis

Hye Sung Kim[1,] Cheol Lee[2], Woo Ho Kim[2], Young Hee Maeng[1,*],Bo Gun Jang[1,*]

[1]Department of Pathology, Jeju National University School of Medicine, Jeju, 690-767, Korea

[2]Department of Pathology, Seoul National University College of Medicine, Seoul, 110-799, Korea

[*]Authors share co-corresponding authorship.

Correspondence

Bo Gun Jang, MD, PhD
Department of Pathology, Jeju National University School of Medicine
Aran 13gil 15, Jeju city, Jeju 690-767, Korea
Tel.:82-64-717-1413; Fax: 82-64-717-1494; E-mail: bgjang9633@gmail.com

Supplementary Table S1 Overview of the intestinal stem cell markers in mouse small intestine and colon

Cell type	Marker	Lineage tracing		Expression (by reporter gene or RNA ISH)	
		Small Intestine	Colon	Small Intestine	Colon
**Crypt base columnar cells**	Lgr5[1]	+	+	Crypt base	Crypt base
	Ascl2[2]	Unknown	Unknown	Crypt base	+*
	Smoc2[3]	+	Unknown	Crypt base	Unknown
**+4 cells**	Bmi1[4-6]	+	–	+4 position	–
	Hopx[7]	+	Unknown	+4 position	Unknown
	Lrig1[8]	+	+	+2 to +5 position	Crypt base
	Tert[9]	+	+	+4 position	+*
**Tuft cells**	Dclk1[10]	–	–	Throughout the crypt-villus axis	Throughout the crypts
**Progenitor cells**	Prom1[11,12]	+	Unknown	Lower half of crypts	Unknown
	Ephb2[13]	Unknown	Unknown	Lower half of crypts#	Unknown
	Msi1[14]	Unknown	Unknown	Lower half of crypts	Unknown

ISH: in situ hybridization. *Expression was only observed by RT-PCR analysis. #Protein expression was detected by immunohistochemistry.

References

1 Barker, N. *et al.* Identification of stem cells in small intestine and colon by marker gene Lgr5. *Nature* **449**, 1003-1007 (2007).

2 van der Flier, L. G. *et al.* Transcription factor achaete scute-like 2 controls intestinal stem cell fate. *Cell* **136**, 903-912 (2009).

3 Muñoz, J. *et al.* The Lgr5 intestinal stem cell signature: robust expression of proposed quiescent '+ 4'cell markers. *The EMBO journal* **31**, 3079-3091 (2012).

4 Sangiorgi, E. & Capecchi, M. R. Bmi1 is expressed in vivo in intestinal stem cells. *Nature genetics* **40**, 915-920 (2008).

5 Yan, K. S. *et al.* The intestinal stem cell markers Bmi1 and Lgr5 identify two functionally distinct populations. *Proceedings of the National Academy of Sciences* **109**, 466-471 (2012).

6 Tian, H. *et al.* A reserve stem cell population in small intestine renders Lgr5-positive cells dispensable. *Nature* **478**, 255-259 (2011).

7 Takeda, N. *et al.* Interconversion between intestinal stem cell populations in distinct niches. *Science* **334**, 1420-1424 (2011).

8 Powell, A. E. *et al.* The pan-ErbB negative regulator Lrig1 is an intestinal stem cell marker that functions as a tumor suppressor. *Cell* **149**, 146-158 (2012).

9 Montgomery, R. K. *et al.* Mouse telomerase reverse transcriptase (mTert) expression marks slowly cycling intestinal stem cells. *Proceedings of the National Academy of Sciences* **108**, 179-184 (2011).

10 Nakanishi, Y. *et al.* Dclk1 distinguishes between tumor and normal stem cells in the intestine. *Nature genetics* **45**, 98-103 (2013).

11 Zhu, L. *et al.* Prominin 1 marks intestinal stem cells that are susceptible to neoplastic transformation. *Nature* **457**, 603-607 (2009).

12 Snippert, H. J. *et al.* Prominin-1/CD133 marks stem cells and early progenitors in mouse small intestine. *Gastroenterology* **136**, 2187-2194. e2181 (2009).

13 Merlos-Suárez, A. *et al.* The intestinal stem cell signature identifies colorectal cancer stem cells and predicts disease relapse. *Cell stem cell* **8**, 511-524 (2011).

14 Maria Cambuli, F., Rezza, A., Nadjar, J. & Plateroti, M. Brief report: Musashi1-eGFP mice, a new tool for differential isolation of the intestinal stem cell populations. *Stem Cells* **31**, 2273-2278 (2013).

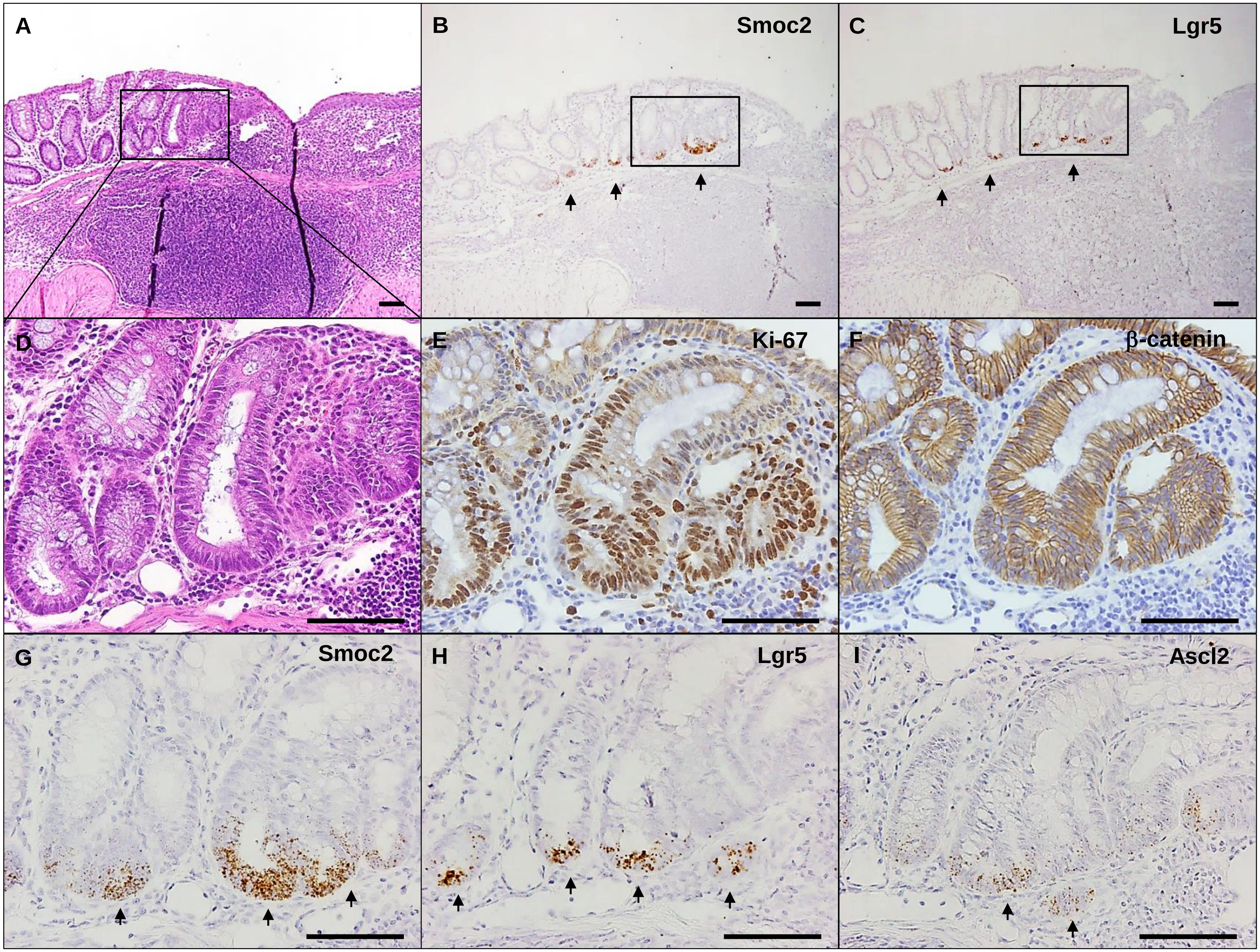

Supplementary Figure S1. Crypt base columnar (CBC) stem cell markers in the regenerative glands. (**A**) H&E stained image of the regenerative glands adjacent to ulcer. Low magnification of RNA in situ hybridization (ISH) for Smoc2 (**B**) and Lgr5 (**C**). High magnification image of regenerative glands (**D**) and immunohistochemistry for Ki-67 (**E**) and β-catenin (**F**). (**G**–**I**) RNA ISH for Smoc2, Lgr5, and Ascl2. G and H are the magnified images of boxed areas in B and C, respectively. CBC stem cell are indicated by arrows. H&E, hematoxylin and eosin. Scale bar: 50μm.

A

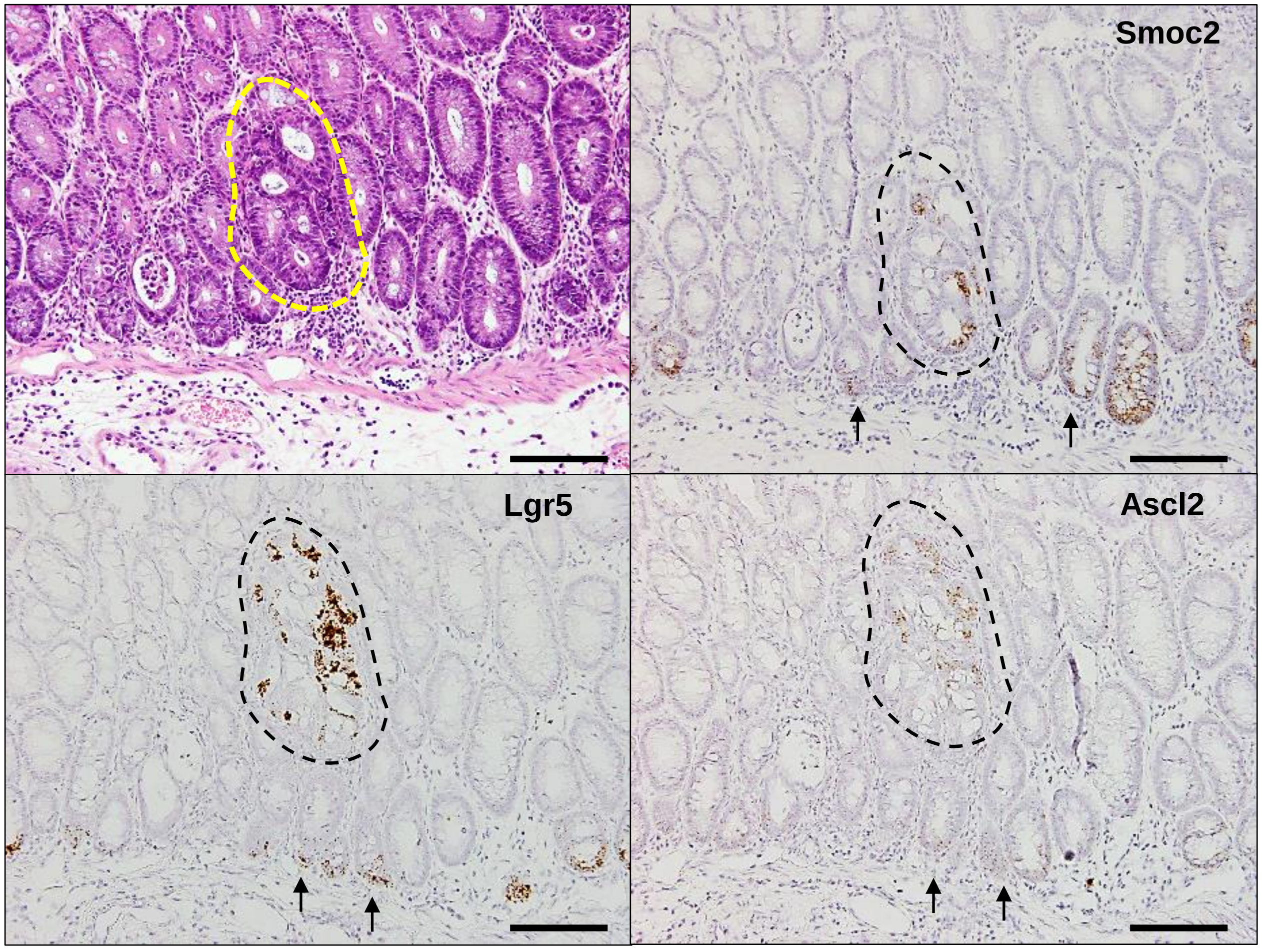

B

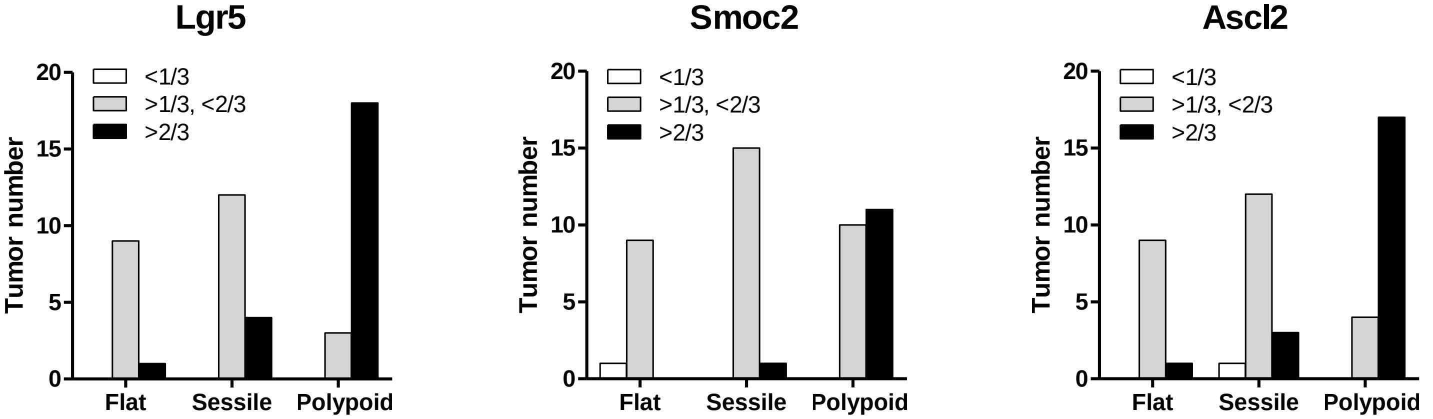

Supplementary Figure S2. Distribution of crypt base columnar (CBC) stem cell markers colitis-associated dysplastic lesions and cancers. (**A**) An aberrant crypt focus indicated by dotted line. Disorganized expression of Smoc2, Lgr5, and Ascl2 by RNA in situ hybridization. Arrows indicate normal CBC stem cells restricted to the bases of the crypts. (**B**) Number of tumors according to gross appearance and distribution of CBC stem cell markers. (Flat: n = 10, Sessile: n = 16, Polypoid: n = 21). < 1/3: lower than one-third of the tumor height; > 1/3, < 2/3: higher than one-third, but lower than two-thirds of the tumor height; > 2/3: higher than two-thirds of the tumor height. Scale bar: 100μm.

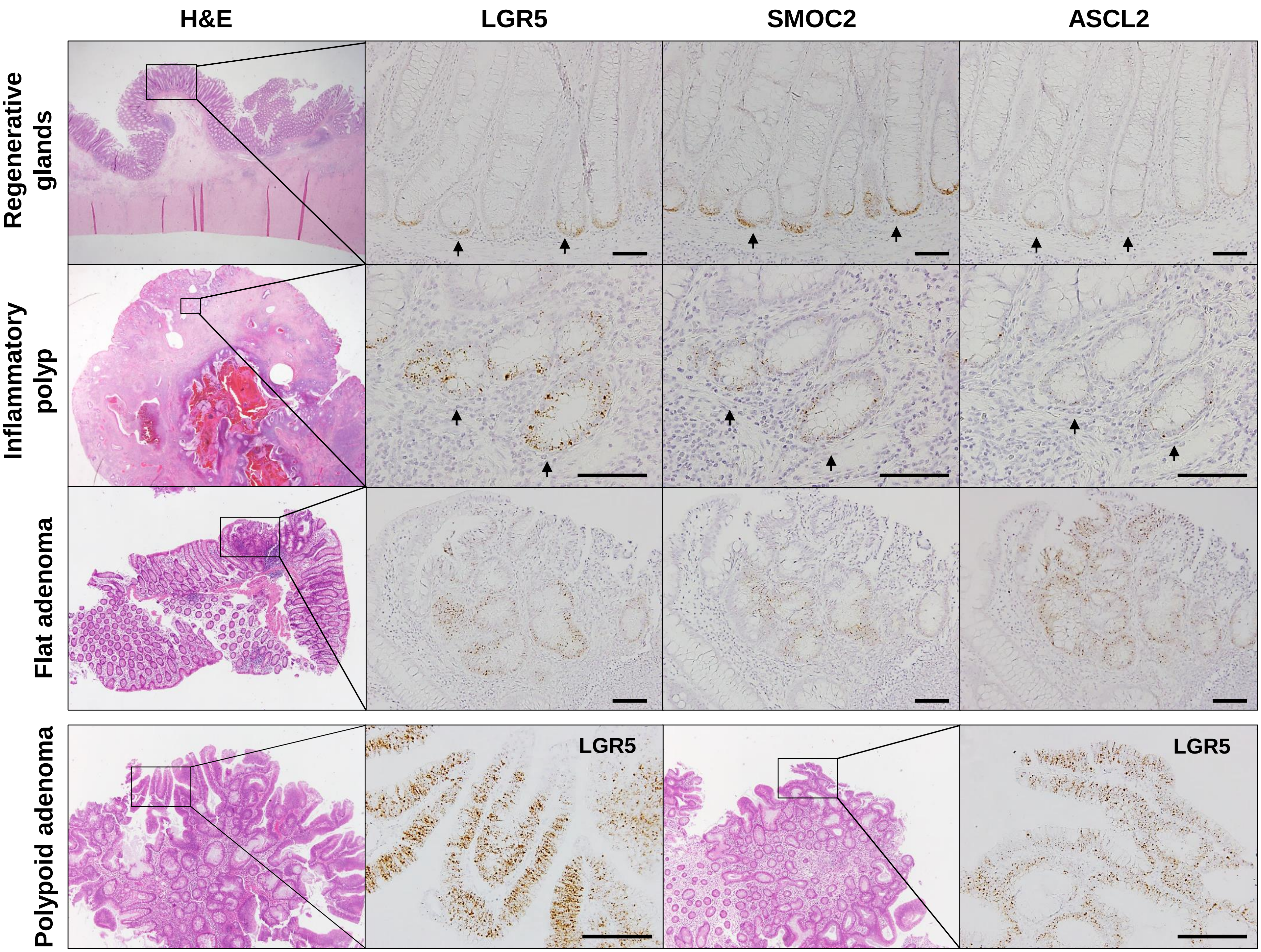

Supplementary Figure S3. Crypt base columnar (CBC) stem cell markers in ulcerative colitis-associated lesions. Representative hematoxylin and eosin-stained images of regenerative glands (RG), inflammatory polyp, flat and polypoid low-grade adenomas from 5 ulcerative colitis patients are shown. Expression of CBC stem cell markers (LGR5, SMOC2, and ASCL2) in RG and Inflammatory polyp are confined to the crypt bases. (Arrows indicate the stem cells at the base of crypts). Flat adenomas exhibit a diffuse expression of CBC stem cell markers. Polypoid adenomas show a strong expression of LGR5 at the upper part of tumor glands. Scale bars: 50μm

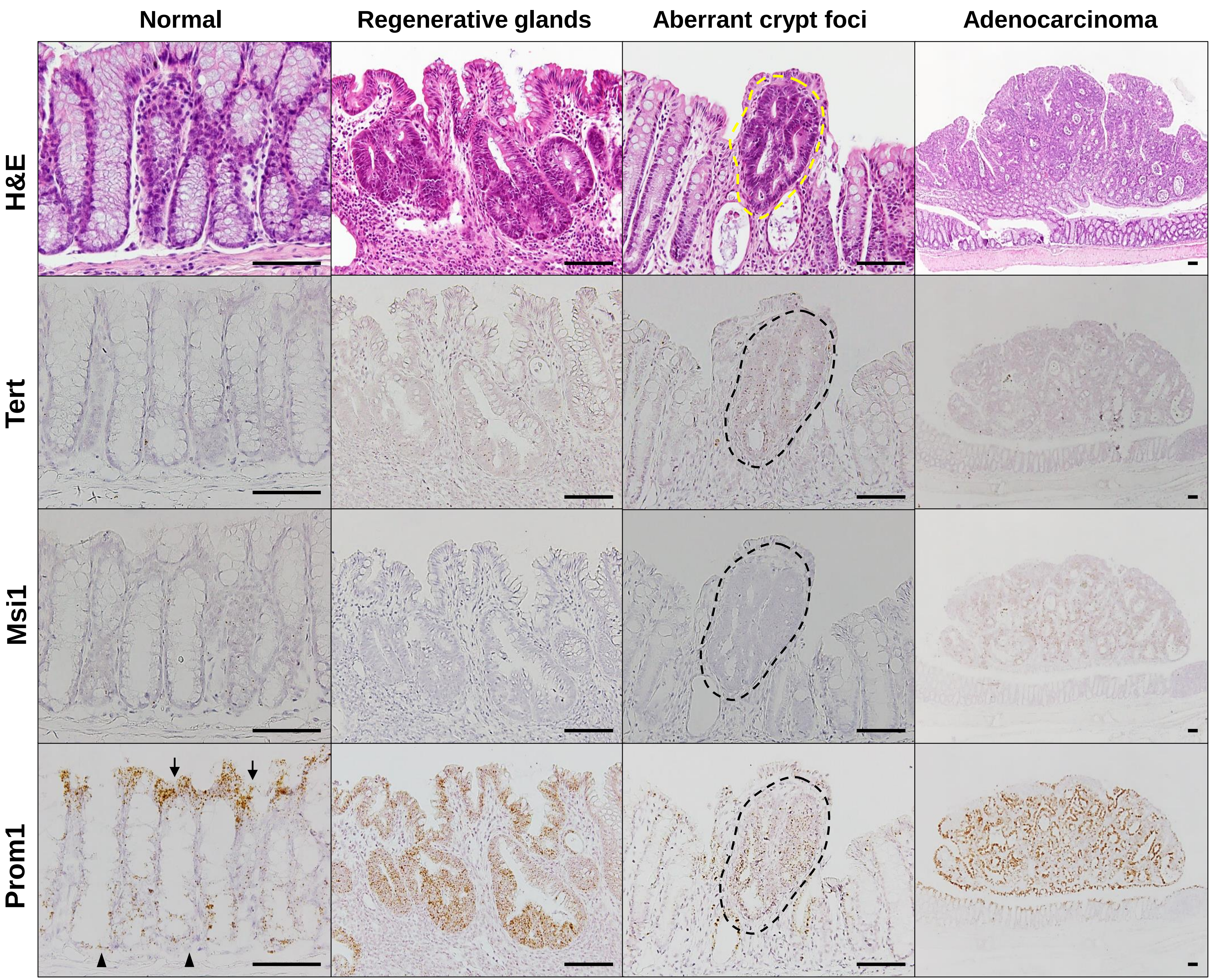

Supplementary Figure S4. RNA in situ hybridization (ISH) for Tert, Msi1 and Prom1 in colitis-associated lesions. Representative hematoxylin and eosin-stained images of normal crypts, regenerative glands (RG), aberrant crypt foci (indicated by yellow and black dotted lines), and adenocarcinoma. Expression of Tert and Msi1 is negligible in the normal crypts and RG and is very low in ACF and adenocarcinoma. Prom1 expression is strong in normal crypts and RG as well as aberrant crypt foci and adenocarcinoma. Arrows indicate the high Prom1 expression in the surface epithelial cells and arrow heads mark Prom1-positive cells at the base of crypts. Scale bars: 50μm
